# Determination, expression and characterization of an UDP-N-acetylglucosamine:α-1,3-D-mannoside β-1,2-N-acetylglucosaminyltransferase I (GnT-I) from the Pacific oyster, *Crassostrea gigas*

**DOI:** 10.1007/s10719-024-10148-9

**Published:** 2024-04-01

**Authors:** Julia Thoma, Reingard Grabherr, Erika Staudacher

**Affiliations:** 1 Department of Chemistry (DCH), University of Natural Resources and Life Sciences, Vienna, Austria; 2 Department of Biotechnology (DBT), University of Natural Resources and Life Sciences, Vienna, Austria

**Keywords:** UDP-N-acetylglucosamine:α-1,3-D-mannoside β-1,2-N-acetylglucosaminyltransferase I, GnT-I, *Crassostrea gigas*, Mollusca, N-glycosylation, Glycosyltransferase

## Abstract

Molluscs are intermediate hosts for several parasites. The recognition processes, required to evade the host’s immune response, depend on carbohydrates. Therefore, the investigation of mollusc glycosylation capacities is of high relevance to understand the interaction of parasites with their host. UDP-N-acetylglucosamine:α-1,3-D-mannoside β-1,2-N-acetylglucosaminyltransferase I (GnT-I) is the key enzyme for the biosynthesis of hybrid and complex type N-glycans catalysing the transfer of N-acetylglucosamine from UDP-N-acetylglucosamine to the α-1,3 Man antenna of Man_5_GlcNAc_2_. Thereby, the enzyme produces a suitable substrate for further enzymes, such as α-mannosidase II, GlcNAc-transferase II, galactosyltransferases or fucosyltransferases. The sequence of GnT- I from the Pacific oyster, *Crassostrea gigas*, was obtained by homology search using the corresponding human enzyme as the template. The obtained gene codes for a 445 amino acids long type II transmembrane glycoprotein and shared typical structural elements with enzymes from other species. The enzyme was expressed in insect cells and purified by immunoprecipitation using protein A/G-plus agarose beads linked to monoclonal His-tag antibodies. GnT-I activity was determined towards the substrates Man5-PA, MM-PA and GnM-PA. The enzyme displayed highest activity at pH 7.0 and 30 °C, using Man5-PA as the substrate. Divalent cations were indispensable for the enzyme, with highest activity at 40 mM Mn^2+^, while the addition of EDTA or Cu^2+^ abolished the activity completely. The activity was also reduced by the addition of UDP, UTP or galactose. In this study we present the identification, expression and biochemical characterization of the first molluscan UDP-N-acetylglucosamine:α-1,3-D-mannoside β-1,2-N-acetylglucosaminyltransferase I, GnT-I, from the Pacific oyster *Crassostrea gigas*.

**Graphical abstract:**

Illustration of GnT-I activity. (a) Transfer of GlcNAc to Man5-PA, creating Man5GlcNAc3-PA. (b) Transfer of GlcNAc to MM-PA, creating MGn-PA. (c) Transfer of GlcNAc to GnM-PA, creating GnGn-PA. Blue squares represent N-acetylglucosamine, green cycles depict mannose. Graphic illustration of N-glycans were created using bioRENDER.

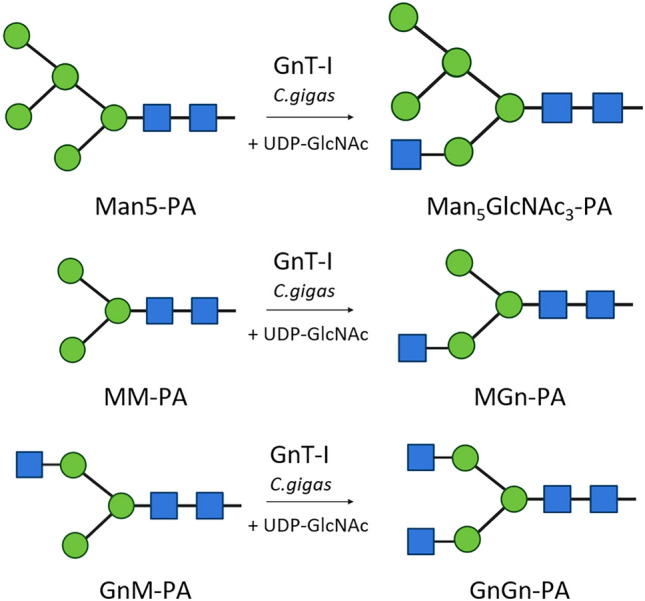

**Supplementary Information:**

The online version contains supplementary material available at 10.1007/s10719-024-10148-9.

## Introduction

Molluscs play an important role in the life cycle of parasites as they are frequently intermediate hosts for the developmental stages of parasites. For example, the fresh-water snails *Biomphalaria glabrata* and *Lymnaea stagnalis* are hosts for *Schistosoma mansoni* and *Trichobilharzia* species, respectively [1–4]. The land snail *Achatina fulica* is host for *Angiostrongylus cantonensis*, the causative agent of human eosinophilic encephalitis [5] and *Crassostrea virginica* is often intermediate host for *Perkinsus marinus* [6]. The selection of the parasite´s intermediate host is highly specific and may depend on the ability to avoid the internal defence of the snails. Glycosylation patterns play an important role in many recognition processes and seem to have a high impact in host-parasite interactions [7]. Presumably to enable invasion and development of the parasite, molluscs and parasites share some structural features of their N- and O-glycans [8, 9]. Therefore, analysis of the glycosylation pathway of molluscs is essential for a better understanding of parasite-host interactions and to advance research into new drug production.

Genomic data facilitate the identification of glycoenzymes and are commonly used to express recombinant enzymes. However, there is only limited genetic information available for molluscs [10], which makes homology searches for putative enzymes extremely challenging. Genetic information is available among others for *Crassostrea gigas*, and *Biomphalaria glabrata*. *Biomphalaria glabrata* is a fresh-water snail that is geographically distributed in South America and Africa. It is the commonly used model organism in snail research due to its close association with schistosomiasis [11, 12]. *Crassostrea gigas* also known as the Pacific oyster, is of culinary benefit to mankind, as it is the most widely consumed oyster in the world. It is cultivated on an industrial scale in Korea, Japan and China. Worldwide it is especially valued due to its nutritional and bioactive properties [13].

The biosynthesis of N-glycans is a two-step process occurring in the rough ER and the Golgi apparatus. In a first step, a highly conserved 14 sugar glycan (Glc_3_Man_9_GlcNAc_2_) linked to a dolichol-phosphate anchor (Dol-P) is transferred “en bloc” to an asparagine within a specific consensus sequence (Asn-X-Ser/Thr) of a nascent peptide chain. In a second step, the N-glycan is modified within the ER by mannosidases and further processed by species-specific glycosidases and glycosyltransferases in the Golgi [14–16]. In general, the processed N-glycans can be classified into three different types, all sharing the conserved pentasaccharide core Man_3_GlcNAc_2_: (1) oligomannosidic type, in which only Man residues extend the core structure; (2) hybrid type, Man extends the Manα1-6 arm and at least GlcNAc extends the Manα1-3 arm and (3) complex type, in which at least GlcNAc is attached to both antennae of the core structure [16].

The key enzyme for the biosynthesis of hybrid and complex N-glycans is UDP-N-acetylglucosamine:α-1,3-D-mannoside β-1,2-N-acetylglucosaminyltransferase I, GnT-I (EC 2.4.1.101), located in the medial-Golgi. GnT-I initiates the synthesis of complex and hybrid type N-glycan structures by catalysing the transfer of N-acetylglucosamine from UDP-N-acetylglucosamine (UDP-GlcNAc) to the Manα1-3 arm of Man_5_GlcNAc_2_. Subsequently, the terminal Man-residues are removed by α-mannosidase II from the Manα1-6 arm. Once these sugars are removed, GnT-II attaches GlcNAc onto the Manα1-6 arm and further glycosyltransferases can modify the N-glycan [17].

GnT-I´s function and characteristics have been intensely studied in vertebrates, such as human [18], rat [19], rabbit [20, 21], bovine [22], *Xenopus laevis* [23], and in different plant species [24–26]. In vertebrates, the prior GnT-I activity is strictly required for the further action of α-mannosidase II and GnT-II. In mammals, deletion of the GnT-I enzyme prevents the formation of hybrid or complex type N-glycans and results in severe developmental abnormalities, as shown for mouse embryos [27].

GnT-I is also the key enzyme for advanced glycan modification in invertebrates. The enzymes of worms (*Caenorhabditis elegans*) and insects (*Drosophila melanogaster*) were previously characterized and cloned [28–30]. Unlike vertebrates, invertebrates produce mainly paucimannosidic N-glycan, with only a small number of complex or hybrid types. Paucimannosidic glycans are small highly modified versions of the core structure Man_3_GlcNAc_2_. However, the prior action of GnT-I is still essential for modification, even when the transferred GlcNAc residue is often removed afterwards by a β-N-acetylglucosaminidase activity [31, 32]. Even less information is available about N-glycosylation in mollusc species [33]. The only characterized GnT-I was purified from the connective tissue of *Lymnaea stagnalis* snails, but not cloned and expressed recombinantly [34]. GnT-I is an essential enzyme for normal development in both vertebrates and invertebrates [35]. Therefore, it is a key factor for the biosynthesis of N-glycans which are relevant for the interaction of parasites and their hosts. In this study we present, for the first time, the cloning, expression and biochemical characterization of a GnT-I from mollusc origin, the Pacific oyster *Crassostrea gigas*.

## Methods

Q5 / Taq DNA polymerases, restriction enzymes and T4 ligase, were purchased from New England Biolabs (Frankfurt, Germany). All enzymes were used according to the supplier's instructions. Primers and gblock gene fragment were synthesized commercially at Sigma-Aldrich (Vienna, Austria) and Integrated DNA Technologies (Leuven, Belgium), respectively. pACEBac1 vector was purchased from Geneva Biotech (Genève, Switzerland). All other chemicals and molecular biology reagents were of the highest quality available purchased from Sigma-Aldrich (Vienna, Austria), Merck (Darmstadt, Germany), Roth (Karlsruhe, Germany), Honeywell (Vienna, Austria) and ThermoFisher Scientific (Bonn, Germany) unless indicated otherwise.

Transformed electrocompetent *E. coli* cells—Neb5α (NEB, Frankfurt, Germany) were spread on Lysogeny Broth (LB) agar plates containing 15 µg/ml gentamycin and incubated overnight at 37 °C. Transformed electrocompetent *E. coli* cells – DH10EMBacY cells (Geneva Biotech—Genève, Switzerland) were cultivated on LB-agar plates containing 15 µg/ml gentamycin, 50 µg/ml kanamycin, 10 µg/ml tetracycline, 50 µg/ml IPTG, 100 µg/ml X-Gal and incubated for 2 days at 37 °C. Electroporation was done using a MicroPulser from BIORAD. *Spodoptera frugiperda* cells – Sf9 (ATCC, Manassas Virginia) were grown in SFM4Insect media with L-Glutamine (HyClone Cytiva – Vienna, Austria) and kept at 27 °C [36]. Viable cell numbers were determined using the Vi-Cell™ XR cell viability analyser (Beckman Coulter – Vienna, Austria).

Substrates for GnT-I (Man5, Man6, MM, MMXF^3^, GnM, MGn^1^; Fig. S1) were prepared as previously described [37–39] and labelled with 2-aminopyridine (PA) according to [38].

pNP-Sugars were from Sigma-Aldrich (Vienna, Austria), mono- and disaccharides obtained from Sigma-Aldrich were labelled with 2-amino benzoic acid (AA) according to [40].

### Identification of the GnT-I gene sequences from *C. gigas*

Putative GnT-I enzymes from *C. gigas* were obtained by BLASTp search (NCBI), within the Mollusca database (taxid: 6447), using the human MGAT1 (UniProt Ref. Seq.: P26572.2) as the template. Four isoforms (X1-X4) were identified within the *C. gigas* species (48.89%, 49.33%, 49.44%, 49.89% similarity to human MGAT1, Fig. S2). The four isoforms derived from automated computational analysis from a whole genome shotgun sequence (NC_047560.1) and were annotated by The Roslin Institute at University of Edinburgh (Easter Bush Campus, United Kingdom) using the gene prediction method “Gnomon”.

Isoforms X3 and X4 were missing exon 2 and isoforms X2 and X4 showed shortened versions of exon 3. Apart from that all isoforms were identical. Isoform X1 from *C. gigas* (NCBI Ref. Seq.: XP_034321804.1, 48.89% identity to human MGAT1) was chosen for expression in Sf9 insect cells, because of its highest number of coding exons and the longest amino acid sequence. The gene sequence was synthetized by IDT (Leuven, Belgium) with codon optimization for insect cells by the company provided IDT codon optimization tool. Alignment studies were performed with the active human MGAT1 and other species such as *C. elegans*, *D. melanogaster* and *B. glabrata* using Clustal Omega.

### Expression of the full-length and truncated versions of the putative GnT-I protein from *C. gigas*

The putative GnT-I isoform X1 of *C. gigas* (GnT-I X1, NCBI Ref. Seq.: XP_034321804.1) was chosen and expressed as a full-length version (membrane-bound protein) and as a truncated version starting at M24 (ΔR2-Y23 GnT-I for *C. gigas*) after the putative transmembrane domain (soluble protein). C- or N-terminal His-tags were added to the sequence for purification. As the IDT synthesized gblock already contained a C-terminal His-tag, the N-terminal His-tag and both restriction sites (EcoRI and XbaI) for cloning into pACEBac1 were added to the sequence via primers (Table 1).

**Table 1:** Primer sequences for the gene amplification of the full-length and truncated protein versions

**Primer name**	**Sequence (5′→ 3’)**	**Restriction site**
GnTI_HIS_EcoRI_fwd	GATGATGAATTCATGCACCATCATCA TCACCATGGCTCAGGTAGACGCA AGCATCTGG	EcoRI
GnTI_EcoRI_fwd	GATGATGAATTCATGAGACGCA AGCATCTGG	EcoRI
tGnTI_HIS_EcoRI_fwd	GATGATGAATTCATGCACCATCAT CATCACCATGGCTCAGGTATGTTGG TATCCAGAAACCC	EcoRI
tGnTI_EcoRI_fwd	GATGATGAATTCATGATGTTGGTAT CCAGAAACCC	EcoRI
univ_GnTI_HIS_XbaI_rev	GATGATTCTAGACTAATGATGGTGAT GATGGTG	XbaI
univ_GnTI_XbaI_rev	GATGATTCTAGACTATGACCACTTTGG ATCATATCC	XbaI

Restriction sites (EcoRI and XbaI) are underlined in the primer sequence

The recombinant genes were PCR amplified by using the according forward and reverse primers. The purified PCR fragment was ligated with the pACEBac1 vector in a 5:1 ratio. The recombinant constructs were transformed to Neb5α (NEB, Frankfurt, Germany) and the correct insertion and gene sequence were verified by sanger sequencing (Microsynth – Vienna, Austria). Approximately 1 ng of the purified construct was used for integration into a MultiBac genome via Tn7 transposition in DH10EMBacY cells (Geneva Biotech, Genève, Switzerland). Midiprep of DH10EMBacY was performed by modifying the NucleoSpin Plasmid EasyPure kit protocol (Macherey–Nagel, Dueren, Germany) according to [41]. 5 µg of the recombinant GnT-I constructs were transfected to Sf9 insect cells using the FuGENE HD Transfection Reagent (Promega - Walldorf, Germany). The cells were centrifuged resulting in a supernatant and a cell pellet. The different GnT-I protein versions were extracted from cell pellet fraction via the I-PER Cell Protein Extraction Reagent (ThermoFisher Scientific - Bonn, Germany), referred as “lysate fraction” later on. Further purification of the protein was achieved through immunoprecipitation using protein A/G-plus agarose beads (CALBIOCHEM – San Diego, United States) linked to mouse anti Penta Histidine Tag:HRP monoclonal antibodies (BIORAD - Vienna, Austria). For the GnT-I activity tests, the enzyme was not eluted from the protein A/G-plus agarose beads, because activity was lost by the suggested acidic elution buffer (0.2 M acetate buffer, pH 3.5) and could not be recovered. Instead, the enzyme was kept linked to the beads and resuspended in MES buffer (0.4 M, pH 6.5). The purified recombinant constructs were analysed by SDS-Page and Western blot using mouse anti Penta Histidine Tag:HRP monoclonal antibodies (1:2500, BIORAD - Vienna, Austria) followed by alkaline phosphatase conjugated anti-mouse IgG from goat (1:4000, Sigma-Aldrich, Vienna, Austria) [42].

### Determination of GnT-I activity

Analysis of GnT-I activity was performed in 25 µl containing 12.5 µl (about 1.25 µg) of purified enzyme (linked to protein A/G-plus agarose beads, resuspended in 0.4 M MES buffer pH 6.5), substrate (12 μM), Mn^2+^ (24 mM), UDP-GlcNAc (50 nmol), ATP (1.6 mM) and NaCl-solution (0.09%). The assay was incubated at 37 °C for 2 h. The product was separated on a reverse-phase C18 column (ODS HypersilTM, 250 × 4 mm, ThermoFisher Scientific - Bonn, Germany) with solvent A composing of 0.1 M NH_4_Ac, pH 4.0 in H_2_O and solvent B containing 30% MeOH in H_2_O. Elution was achieved by a linear gradient with solvent B from 0–30% in 30 min, at a flow rate of 1.5 ml/min. Quantification was achieved by peak integration after fluorescence detection at 320 nm excitation and 400 nm emission [38].

For analysis of the enzyme’s biochemical parameters, the standard conditions of the incubation assay, using Man5-PA as the substrate, were modified as follows. For the determination of cation requirement, the standard assay was carried out without any cation addition (H_2_O) or in presence of 24 mM of EDTA, Mn^2+^, Mg^2+^, Ca^2+^, Co^2+^, Cu^2+^, Ni^2+^ or Ba^2+^. Chemical stability of the enzyme, optimal storage conditions, and pH-optimum were processed according to [43]. For storage stability in chemicals, the enzyme was incubated for 16 h in 10% or 20% of methanol, acetonitrile, glycerol, or imidazole [50 mM or 100 mM]. For inhibition studies the standard assay was performed in the presence of 0.1% UMP, UDP, UTP, Gal, Glc, GlcNAc or Man. A time course (up to 24 h) was carried out by measuring the transfer of GlcNAc to Man5-PA.

Each assay was performed with two biological replica (different expression batches) and the appropriate controls (purified lysate without recombinant GnT-I protein).

### GnT-I substrate specificity

Substrate specificity was analysed with various labelled sugars and glycans using standard assay conditions.

4-Nitrophenyl-sugars: pNP-α-Man, pNP-β-Man, pNP-α-GalNAc, pNP-β-GalNAc, pNP-α-Gal, pNP-β-Gal, pNP-α-GalNAcβ1,3Gal (pNP-Galacto-N-biose, O-glycan core 1) and pNP-Glcβ1,4Gal (pNP-Lactose). Separation of pNP-sugars was done on reverse-phase HPLC (ODS HypersilTM, 250 × 4.6 mm, ThermoFisher Scientific - Bonn, Germany) with solvent A composing of 0.1 M ammonium acetate, pH 6.0 and solvent B containing 50% (v/v) acetonitrile in H_2_O. Elution was achieved by a linear gradient of solvent B from 5–50% in 30 min, at a flow rate of 1 ml/min. Quantitative values were obtained by peak integration after UV detection at 280 nm.

2-Amino-benzoic acid labelled sugars: Galβ1,4Glc-AA (Lactose-AA), Galβ1,3GalNAc-AA (Galacto-N-biose-AA, O-glycan core 1), Galβ1,3GlcNAc-AA (Lacto-N-biose-AA), Galβ1,4GlcNAc-AA (N-Acetyllactosamine-AA) and Galβ1,6GlcNAc-AA. Separation of 2-amino-benzoic acid labelled sugars was carried out on reverse-phase HPLC (ODS HypersilTM, 250 × 4 mm, ThermoFisher Scientific - Bonn, Germany) with solvent A: 0.2% (v/v) 1-butylamin, 0.5% (v/v) orthophosphoric acid, 1% (v/v) tetrahydrofuran in H_2_O and solvent B: solvent A/acetonitrile = 50/50 (v/v). The elution was performed by a linear gradient of solvent B from 5–100% in 23 min, at a flowrate of 1 ml/min. Quantification was done by peak integration after fluorescence detection at ex/em 360 nm/425 nm [40].

2-Aminopyridine labelled N-glycans: MM-PA, MMXF^3^-PA, Man6-PA, GnM-PA and MGn-PA. Separation of 2-aminopyridine labelled N-glycans was carried out as described for the standard assay.

### MALDI-TOF MS analysis

MALDI-TOF MS analysis was performed on an Autoflex Speed MALDI-TOF (Bruker Daltonics, Germany) equipped with a 1000 Hz Smartbeam.II laser in positive mode using 2% (w/v) dihydroxybenzoic acid in 50% (v/v) acetonitrile as matrix. The spectra were processed with the manufacturer’s software (Bruker Flexanalysis 3.3.80).

## Results

### Identification of putative GnT-I proteins in *C. gigas*

**Fig. 1:**
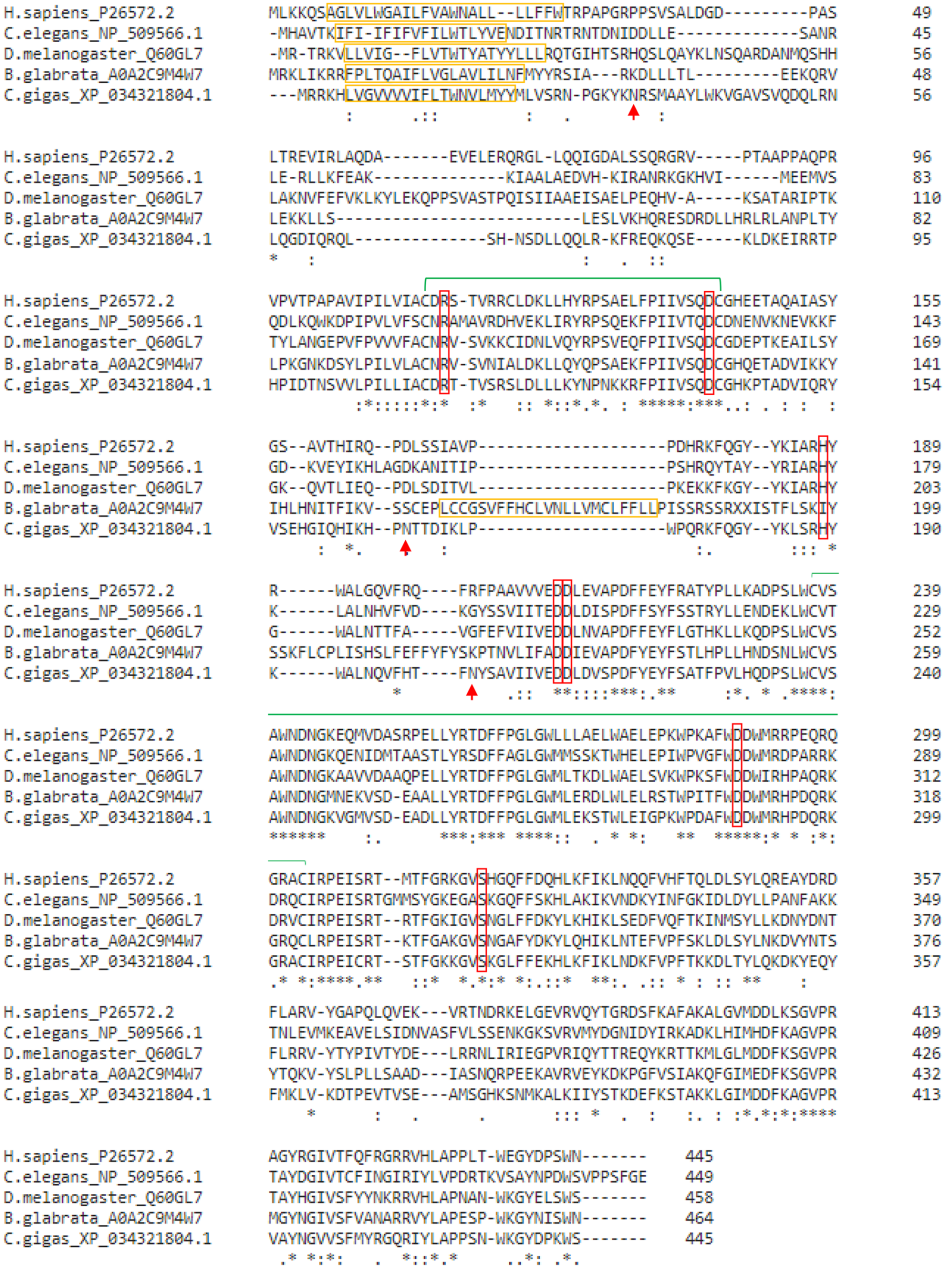
Sequence comparison of GnT-I from different species (*H. sapiens*: P26572.2; *C. elegans*: NP_509566.1; *D. melanogaster*: Q60GL7; *B. glabrata*: A0A2C9M4W7 and *C. gigas*: XP_034321804.1). The transmembrane domains are depicted in yellow boxes, the 7 binding sites (substrate and Mn^2+^ binding) in red boxes and disulfide bridges by green bars. Putative N-glycosylation sites (shown solely for *C. gigas*) are indicated by red arrows

Gene sequences for the GnT-I from *C. gigas* were obtained by a BLASTp search using the human MGAT1 sequence (UniProt Ref. Seq.: P26572.2) as the template. Overall, we identified 4 isoforms, all located at chromosome 2 at the gene loci LOC105346421. The isoforms X1 and X2 consisted of 12 coding exons, while the isoforms X3 and X4 consisted of 11 coding exons (missing exon 2). Isoform X1 (445 AA, NCBI Ref. Seq.: XP_034321804.1) was chosen for further experiments, because it had the most complete sequence. It showed sequence homology with previously identified invertebrate GnT-I proteins (*C. elegans* 41.07%, *D. melanogaster* 44.61%; Fig. 1).

The GnT-I from *C. gigas* displayed a type II-transmembrane domain (amino acid 6 – 25), predicted by TMHMM 2.0 – DTU Health Tech, corresponding to the GnT-Is from other species. The presence of 6 substrate binding sites and the conserved metal binding motive EDD suggested a functional GnT-I enzyme in *C. gigas*. The GnT-I isoform X1 from *C. gigas* contained 3 predicted N-glycosylation sites (NetNGlyc 1.0 – DTU Health Tech, Fig. 1).

### Expression of GnT-I from *C. gigas* and purification of versions in Sf9 insect cells

GnT-I from *C. gigas* was cloned and expressed as full-length protein (membrane-bound protein) as well as a truncated version without the transmembrane domain (soluble protein) (Fig. S3). All constructs were expressed with a C- or N-terminal His-tag in Sf9 insect cells. The full-length recombinant proteins (GnT-I_C, GnT-I_N) consisted of 445 AA and had a molecular weight of approximately 52 kDa, while the truncated versions (tGnT-I_C, tGnT-I_N) contained 423 AA with a molecular weight of around 50 kDa. The full-length protein with C-terminal His-tag (GnT-I_C) and the truncated proteins with either C- or N-terminal His-tag (tGnT-I_C, tGnT-I_N) were detected in the lysate fractions by Western Blot analysis (Fig. S4).

Similar to other glycosyltransferases from mollusc origin, it was a challenge to retain enzyme activity during the purification process. We were successful by purifying the enzyme from the cell lysate by immunoprecipitation using protein A/G-plus agarose beads. The purification by a HisTrap HP affinity column, which should bind a His-tag or by size exclusion chromatography (S200) failed. However, an elution from the agarose beads was not possible due to the required acidic pH, which destroyed the enzyme activity. Therefore, protein characterization was performed with the enzyme bound to the beads.

### Determination of enzyme activity and substrate specificity

**Fig. 2:**
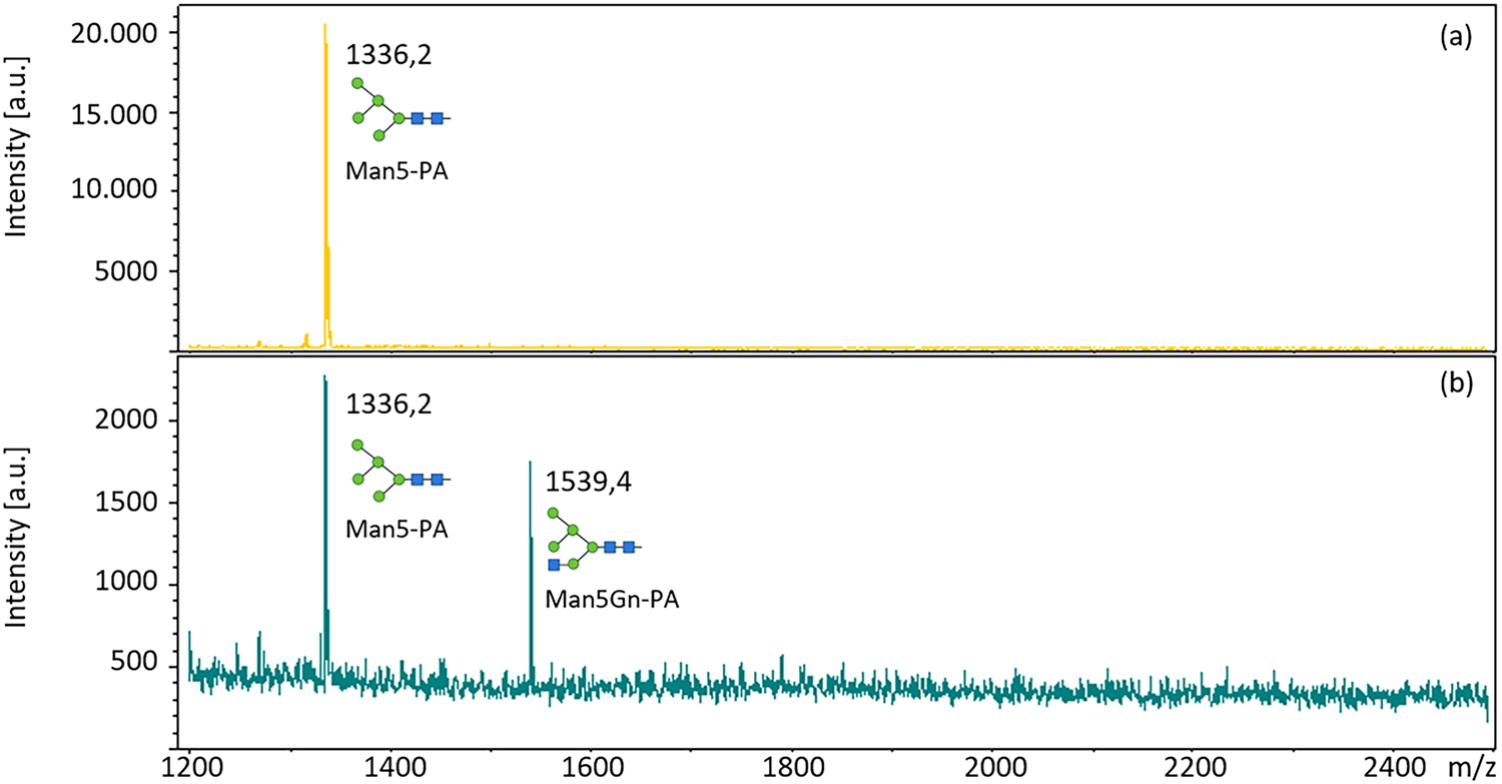
MALDI-TOF analysis of enzyme activity of truncated GnT-I with C-terminal His-tag. **a** Man5-PA substrate; **b** Man5-PA incubated with GnT-I from *C. gigas*; Structures were created using bioRENDER

The transferase activity of the expressed GnT-I constructs was analysed using Man5-PA as the substrate and UDP-GlcNAc as the donor (Fig. 2). No GnT-I activity was detected in the negative controls, which were purified lysate fractions without recombinant expressed GnT-I. Therefore, any GnT-I activity could be excluded from the expression system (Sf9 insect cells). Lysate fractions of all three constructs (GnT-I_C, tGnT-I_C, tGnT-I_N) were active. For further analysis the truncated version of the enzyme with the C-terminal His-tag (tGnT-I_C) was chosen.

**Fig. 3:**
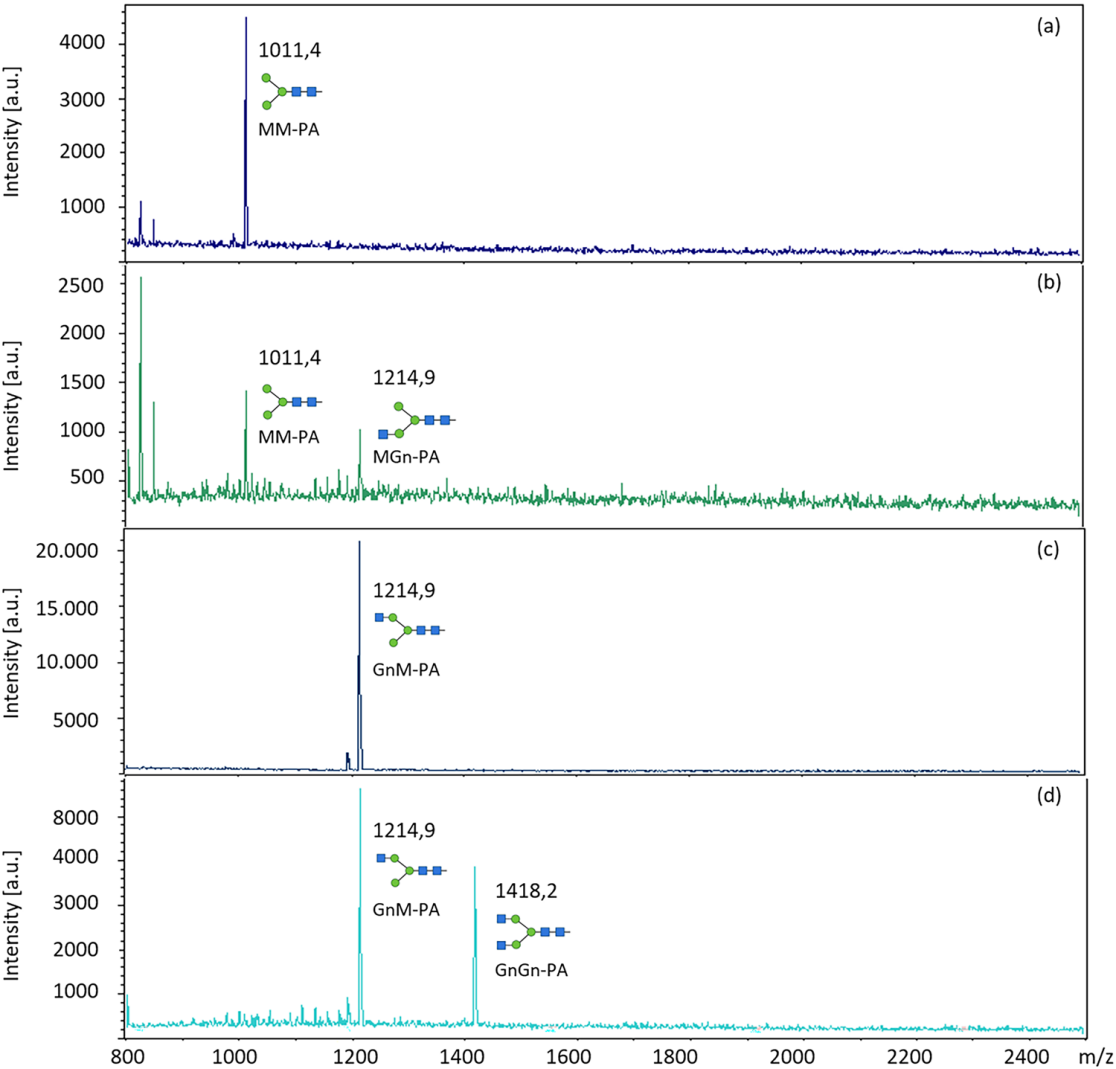
MALDI-TOF analysis of enzyme activity of truncated GnT-I with C-terminal His-tag. **a** MM-PA substrate; **b** MM-PA incubated with GnT-I from *C. gigas*; **c** GnM-PA substrate; **d** GnM-PA incubated with GnT-I from *C. gigas*. Structures were created using bioRENDER

Besides Man5-PA, which was used for protein characterization, the GnT-I substrate specificity was tested on different labelled N-glycans, mono- and di-saccharides (Table 2). Using this selection, the GnT-I from *C. gigas* was only able to transfer GlcNAc to the N-glycans Man5-PA, MM-PA and GnM-PA (Figs. 2 and 3). All other glycans were not suitable substrates.

**Table 2:** Substrate specificity of GnT-I from *C. gigas*

**Substrate**	**GnT-I activity**
Man5-PA	Yes (Fig. 2)
Man6-PA	No
MM-PA	Yes (Fig. 3)
GnM-PA	Yes (Fig. 3)
MGn-PA	No
MMXF^3^-PA	No
Galβ1,4Glc-AA (Lactose-AA)	No
Galβ1,3GalNAc-AA (Galacto-N-biose-AA, O-gylcan core 1)	No
Galβ1,3GlcNAc-AA (Lacto-N-biose-AA)	No
Galβ1,4GlcNAc-AA (N-Acetyllactosamine-AA)	No
Galβ1,6GlcNAc-AA	No
pNP-α-Man	No
pNP-β-Man	No
pNP-α-GalNAc	No
pNP-β-GalNAc	No
pNP-α-Gal	No
pNP-β-Gal	No
pNP-α-GalNAcβ1,3Gal (pNP-Galacto-N-biose, O-glycan core 1)	No
pNP-Glcβ1,4Gal (pNP-Lactose)	No

### Biochemical parameters of recombinant GnT-I from *C. gigas*

**Fig. 4:**
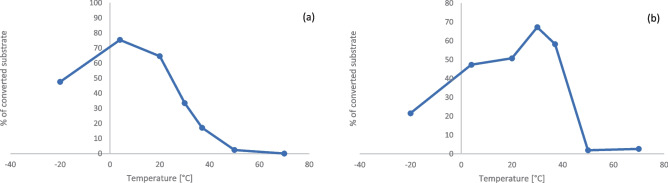
Effect of temperature on GnT-I from *C. gigas*. **a** Optimal storage temperature, **b** Optimal reaction temperature. Data points represent mean values of two biological replica with corresponding standard deviation

The biochemical parameters were determined by HPLC using the *in vivo* substrate Man5-PA. The optimal storage temperature (24 h) of the purified GnT-I was at 4 °C (Fig. 4a) and the optimal reaction temperature (2 h incubation) was at 30 °C (Fig. 4b).

**Fig. 5:**
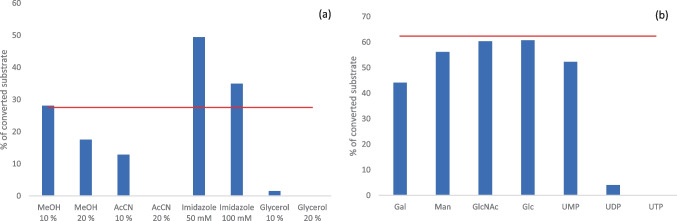
Biochemical properties of GnT-I from *C. gigas*. **a** Stability of the enzyme in presence of chemicals (methanol, acetonitrile, imidazole, glycerol). The red line represents a control without the addition of chemicals. **b** Inhibition studies by adding Gal, Man, GlcNAc, Glc, UMP, UDP or UTP to the assay to a final concentration of 0.1% (w/v). The red line represents a control without the addition of inhibitors. Data points represent mean values of two biological replica with corresponding standard deviation

A storage in methanol (20% v/v), acetonitrile (10% v/v) or glycerol (10% v/v) drastically reduced the enzyme’s activity, while the activity was increased in presence of imidazole (50 mM) (Fig. 5a). Inhibition studies indicated the negative influence of UDP, UTP and galactose on the enzyme’s activity (Fig. 5b). In terms of GnT-I activity over time (time course), we observed GlcNAc transfer to the Man5-PA substrate even after 24 h incubation at 37 °C.

**Fig. 6:**
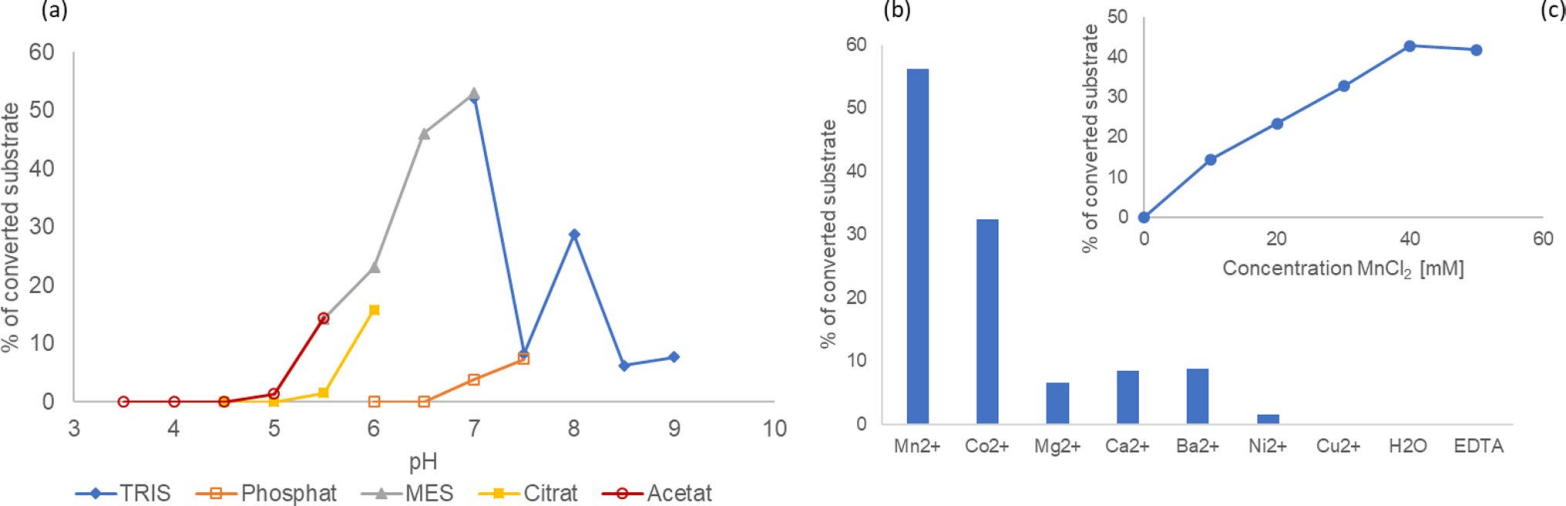
Biochemical properties of GnT-I from *C. gigas*. **a** pH-curve using different buffer salts, **b** Cation requirement and **c** Optimal Mn^2+^ concentration. Data points represent mean values of two biological replica with corresponding standard deviation

The optimal pH was around 7.0 using MES or TRIS as the buffer salts (Fig. 6a). To investigate the importance of divalent cations, the activity assay was carried out without the addition of cations or in presence of EDTA, Mn^2+^, Mg^2+^, Ca^2+^, Co^2+^, Cu^2+^, Ni^2+^ or Ba^2+^. Thereby, the enzyme indicated the need for cations, as the absence of a cation addition or the presence of EDTA vanished the enzymes activity completely. The same negative effect was observed for Cu^2+^. The optimal cation was Mn^2+^ at a concentration of 40 mM. Enzyme activity was also detectable in the presence of Ni^2+^, Mg^2+^, Ca^2+^ and Ba^2+^ but rather low. Besides Mn^2+^, just Co^2+^ had a positive effects on the GnT-I activity (Fig. 6b, c).

## Discussion

Molluscs are a large and evolutionarily very successful phylum of the animal kingdom that can be found in freshwater, marine and terrestrial ecosystems. Some representatives play an important role in the life cycle of parasites, serving as intermediate hosts for their developmental stages. Thereby, glycosylation patterns play an important role in many host-parasite recognition processes. The N- and O-glycan structures known to date suggest broad glycosylation abilities in molluscs, that may advance research into new drug production. It is therefore worth analysing the glycosyltransferases involved in the biosynthesis of these glycans. However, there is only little genetic information available from molluscs, which makes research on these proteins much more difficult.

UDP-N-acetylglucosamine:α-1,3-D-mannoside β-1,2-N-acetylglucosaminyltransferase I (EC 2.4.1.101), also known as GnT-I, is responsible for the transfer of GlcNAc from UDP-GlcNAc to the Manα1-3 arm of Man_5_GlcNAc_2_. Its action is required to produce complex, hybrid or paucimannosidic N-glycans [16, 32]. Although the GnT-I isolated from several plants and animals was cloned and characterized previously, no GnT-I from molluscs had been successfully cloned so far. In general, there is only scattered information available about this enzyme in invertebrates.

Within the *C. elegans* genome, there are 3 isoforms of GnT-I (GLY-12, GLY-13 and GLY-14) encoded on the genome, of which all showed activity towards the physiological N-glycan Man5 [29]. A similar enzyme was described for *D. melanogaster*, which in contrast to *C. elegans*, encodes only a single GnT-I enzyme [30]. By sequence homology search, using the human GnT-I as the template, four putative GnT-I isoforms were revealed within the Pacific oyster, *C. gigas*. Three of them had gaps in their sequence, so the most complete isoform X1 (XP_034321804.1) containing 12 coding exons, was selected for recombinant expression in Sf9 insect cells. Alignment studies showed sequence similarities to previously characterized homologous enzymes of *H. sapiens* (48.89%), *D. melanogaster* (44.61%) and *C. elegans* (41.07%). Similar to many glycosyltransferases, GnT-I possesses a typical DxD-motif mostly present in form of EDD (Fig. 1). In general, the canonical DxD-motif has two Asp residues of which the 1st is relatively variable, and the 2^nd^ (at position 3) is quite well conserved. Thereby, the 2^nd^ Asp is responsible for the interaction with Mn^2+^ [44]. Overall, seven substrate binding sites (including the DxD-motif) were identified by crystallography within the active domain of the rabbit GnT-I [45]. In *C. gigas*, all seven sites were present and identical to *H. sapiens*, *D. melanogaster* and *C. elegans*. Therefore, the functionality of the catalytic domain was given at the genomic level. Furthermore, we identified the presence of a transmembrane domain through TMHMM 2.0 – DTU Health Tech. GnT-I from *C. gigas* belongs to the group of type II transmembrane glycoproteins, presenting the typical features of a short N-terminal cytoplasmic segment, a transmembrane domain, a stem region and a catalytical C-terminal domain [46].

The freshwater gastropod, *B. glabrata* is a fully sequenced model organism known for being an intermediate host for the parasite, *S. mansoni*. Because of its importance to human health and because of the fully available genomic sequence, it is the usual model organism in mollusc research. Therefore, we also tried to clone and express the GnT-I from *B. glabrata* in Sf9 insect cells in parallel to the *C. gigas* enzyme. However, unlike to the *C. gigas* enzyme, the only predicted GnT-I sequence from *Biomphalaria glabrata* was not complete, since two amino acids, position X189 and X190 were not identified. Moreover, we noticed the presence of a second transmembrane domain (F16-F27 and L158-L180), an ADD motif instead of the canonical EDD motif and that the 3rd substrate binding site (histidine) was exchanged by an isoleucine (Fig. 1). The gene encoding this enzyme (Uniprot Ref. Seq.: A0A2C9M4W7, 41.79% identity to human MGAT1) was synthesized by replacing the unknown amino acids with alanine. This modified version of the enzyme was cloned and expressed in Sf9 insect cells but did not show any activity. Which of the conformational changes are responsible for the loss of function of the GnT-I from *B. glabrata* remains to be investigated.

Several studies exist, that focus on the substrate specificity of GnT-I by using substrates such as GnGn, MM, GnM, MGn, Man5, Man_7_GlcNAc_2_ or Man_9_GlcNAc_2_, with the substrates MM, Man5 and GnM having the essential core Manα1,3 arm available [23, 24, 34, 39, 47]. It was generally discovered that GnT-I is able to transfer GlcNAc to MM but prefers the physiological substrate Man5. By using Manα1,3(R1α1,6)Manβ1-R2, it was shown, that the minimum requirement of a Manα1,3Manβ was essential for the enzyme´s activity [48]. Indeed, we also found that GnT-I from *C. gigas* transferred GlcNAc to both Man5-PA and MM-PA N-glycans. Moreover, we were able to detect the attachment of GlcNAc to GnM-PA, indicating an activity after the action of the GnT-II enzyme. Sf-Gn-T I from the insect *Spodoptera frugiperda* showed a similar substrate specificity [49]. It had been assumed that this specificity is able to compensate the trimming by hexosaminidase activity [49]. Since molluscs display paucimannosidic N-glycans similar to insects, this could also be the function in the case of molluscs. Unfortunately, GnM is very rarely tested in the characterization of Gn-T Is and therefore no definitive statement is possible. Similar to the enzyme from *Lymnaea stagnalis* [34], the presence of a β1,2-linked xylose residue at the core Manβ1,4 residue abolished GnT-I activity. Using the selection of substrates given in Table 2, only three N-glycan structures (Man5-PA, MM-PA, GnM-PA) were valuable substrates for the GnT-I from *C. gigas* (Table 2).

Besides the substrate specificity, the enzyme from *C. gigas* also shared biochemical features with other species. The optimal pH-environment of GnT-I was determined to be at 7.5 for *L. stagnalis* [34], between 5.0 to 6.0 for *D. melanogaster* [30], 6.3 for *M. brassicae* [39] and 7.5 to 8.5 for *C. elegans* [28]. Similarly, an optimal pH of 7.0 was found for the GnT-I from *C. gigas* using MES or TRIS as the buffer salts. The use of phosphate buffer showed a clear decrease of the enzyme’s activity. This is because phosphate buffer has a significant effect on glycosyltransferases when using UDP-sugars as substrate. Thereby, phosphate ions have an inhibitory effect by blocking the UDP-binding site of the enzyme [50]. It should be emphasized that acidic pH rapidly abolished the GnT-I activity. Therefore, it was not possible to recover any enzyme activity during purification, even when the contact of the protein with the acidic environment was very short.

The optimal reaction temperature for the GnT-I from *C. gigas* was at 30 °C and in range with *D. melanogaster* (37 °C) [30] and *C. elegans* (20–30 °C) [28]. Moreover, the enzyme was active for up to 24 h at 37 °C. As already shown for other GnT-I enzymes [28, 30, 34, 39, 44], the enzyme from *C. gigas* depended on cations with a high preference for Mn^2+^ And was inhibited by UDP, UTP and galactose.

More than 25 years after the first detection of GnT-I activity in the snail *Lymnaea stagnalis* [34], we have now been able to express this enzyme recombinantly from mollusc origin for the first time. UDP-N-acetylglucosamine:α-1,3-D-mannoside β-1,2-N-acetylglucosaminyltransferase I (GnT-I) from *Crassostrea gigas* shares structural as well as biochemical characteristics with the corresponding enzymes from other phyla. This highly conserved enzyme is the key enzyme for the biosynthesis of complex and hybrid N-glycans, which forms glycan structures that may play an important role in the recognition and interaction processes between parasites and their hosts. Furthermore, a better understanding of the glycosylation capabilities of molluscs may provide insights into their highly successful adaptations and survival strategies.

### Supplementary Information

Below is the link to the electronic supplementary material.

Supplementary file1 (PDF 430 KB)

## Data Availability

No datasets were generated or analysed during the current study.

## Footnotes

1 **Man5**: [Manα1,6(Manα1,3)Manα1,6](Manα1,3)Manβ1,4GlcNAcβ1,4GlcNAc. **Man6**: [Manα1,6(Manα1,3)Manα1,6](Manα1,2Manα1,3)Manβ1,4GlcNAcβ1,4GlcNAc. **MM**: Manα1,6(Manα1,3)Manβ1,4GlcNAcβ1,4GlcNAc. **MMXF**^**3**^: [Manα1,6](Manα1,3)Xylβ1,2Manβ1,4GlcNAcβ1,4(Fuc α1,3)GlcNAc. **MGn**: Manα1,6(GlcNAcβ1,2Manα1,3)Manβ1,4GlcNAcβ1,4GlcNAc. **GnM**: GlcNAcβ1,2Manα1,6(Manα1,3)Manβ1,4GlcNAcβ1,4GlcNAc.
