# Supplementary material for: Determination, expression and characterization of an UDP-N-acetylglucosamine:α-1,3-D-mannoside β-1,2-N-acetylglucosaminyltransferase I (GnT-I) from the Pacific oyster, Crassostrea gigas
Source: Glycoconj J. 2024 Apr 1;41(2):151–62. doi: 10.1007/s10719-024-10148-9 (PMC11065688; doi:10.1007/s10719-024-10148-9)
Supplement: Supplementary file 1 — Supplementary file1 (PDF 430 KB) [file 10719_2024_10148_MOESM1_ESM.pdf]

Thoma et. al – Supplementary Information

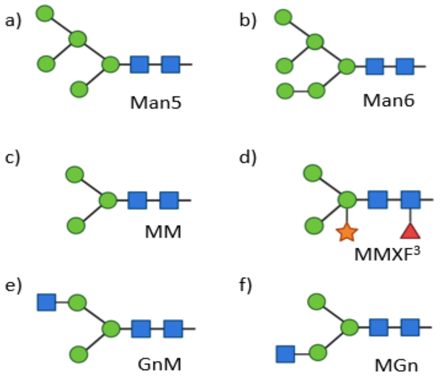

Fig. S1 Graphic illustration of N-glycan GnT-I substrates. (a) Man5, (b) Man6, (c) MM, (d) MMXF^3, (e) GnM and (f) MGn. Blue squares represent N-acetylglucosamine, green cycles illustrate mannose, orange asterisk represents xylose and red triangle depicts fucose. Structures were created using bioRENDER

```
C.gigas_X1_XP_034321804.1      MRRKHLVGVVVVIFLTWNVLMYYMLVSRNPGKYKNRSMAAYLWKVGAVSVQDQLRNLQGD    60
C.gigas_X2_XP_034321805.1      MRRKHLVGVVVVIFLTWNVLMYYMLVSRNPGKYKNRSMAAYLWKVGAVSVQDQLRNLQGD    60
C.gigas_X3_XP_011453276.2      MRRKHLVGVVVVIFLTWNVLMYYMLVSRNPGK------------VGAVSVQDQLRNLQGD    48
C.gigas_X4_XP_011453278.2      MRRKHLVGVVVVIFLTWNVLMYYMLVSRNPGK------------VGAVSVQDQLRNLQGD    48
                               ********************************            ****************

C.gigas_X1_XP_034321804.1      IQRQLSHNSDLLQQLRKFREQKQSEKLDKEIRRTPHPIDTNSVVLPILLIACDRTTVSRS    120
C.gigas_X2_XP_034321805.1      IQRQLSHNSDLLQQLQ---------KLDKEIRRTPHPIDTNSVVLPILLIACDRTTVSRS    111
C.gigas_X3_XP_011453276.2      IQRQLSHNSDLLQQLRKFREQKQSEKLDKEIRRTPHPIDTNSVVLPILLIACDRTTVSRS    108
C.gigas_X4_XP_011453278.2      IQRQLSHNSDLLQQLQ---------KLDKEIRRTPHPIDTNSVVLPILLIACDRTTVSRS    99
                               ***************:         ***********************************

C.gigas_X1_XP_034321804.1      LDLLLKYNPNKKRFPIIVSQDCGHKPTADVIQRYVSEHGIQHIKHPNTTDIKLPWPQRKF    180
C.gigas_X2_XP_034321805.1      LDLLLKYNPNKKRFPIIVSQDCGHKPTADVIQRYVSEHGIQHIKHPNTTDIKLPWPQRKF    171
C.gigas_X3_XP_011453276.2      LDLLLKYNPNKKRFPIIVSQDCGHKPTADVIQRYVSEHGIQHIKHPNTTDIKLPWPQRKF    168
C.gigas_X4_XP_011453278.2      LDLLLKYNPNKKRFPIIVSQDCGHKPTADVIQRYVSEHGIQHIKHPNTTDIKLPWPQRKF    159
                               ************************************************************

C.gigas_X1_XP_034321804.1      QGYYKLSRHYKWALNQVFHTFNYSAVIIVEDDLDVSPDFYEYFSATFPVLHQDPSLWCVS    240
C.gigas_X2_XP_034321805.1      QGYYKLSRHYKWALNQVFHTFNYSAVIIVEDDLDVSPDFYEYFSATFPVLHQDPSLWCVS    231
C.gigas_X3_XP_011453276.2      QGYYKLSRHYKWALNQVFHTFNYSAVIIVEDDLDVSPDFYEYFSATFPVLHQDPSLWCVS    228
C.gigas_X4_XP_011453278.2      QGYYKLSRHYKWALNQVFHTFNYSAVIIVEDDLDVSPDFYEYFSATFPVLHQDPSLWCVS    219
                               ************************************************************

C.gigas_X1_XP_034321804.1      AWNDNGKVGMVSDEADLLYRTDFFPGLGWMLEKSTWLEIGPKWPDAFWDDWMRHPDQRKG    300
C.gigas_X2_XP_034321805.1      AWNDNGKVGMVSDEADLLYRTDFFPGLGWMLEKSTWLEIGPKWPDAFWDDWMRHPDQRKG    291
C.gigas_X3_XP_011453276.2      AWNDNGKVGMVSDEADLLYRTDFFPGLGWMLEKSTWLEIGPKWPDAFWDDWMRHPDQRKG    288
C.gigas_X4_XP_011453278.2      AWNDNGKVGMVSDEADLLYRTDFFPGLGWMLEKSTWLEIGPKWPDAFWDDWMRHPDQRKG    279
                               ************************************************************

C.gigas_X1_XP_034321804.1      RACIRPEICRTSTFGKKGVSKGLFFEKHLKFIKLNDKFVPFTKKDLTYLQKDKYEQYFMK    360
C.gigas_X2_XP_034321805.1      RACIRPEICRTSTFGKKGVSKGLFFEKHLKFIKLNDKFVPFTKKDLTYLQKDKYEQYFMK    351
C.gigas_X3_XP_011453276.2      RACIRPEICRTSTFGKKGVSKGLFFEKHLKFIKLNDKFVPFTKKDLTYLQKDKYEQYFMK    348
C.gigas_X4_XP_011453278.2      RACIRPEICRTSTFGKKGVSKGLFFEKHLKFIKLNDKFVPFTKKDLTYLQKDKYEQYFMK    339
                               ************************************************************

C.gigas_X1_XP_034321804.1      LVKDTPEVTVSEAMSGHKSNMKALKIIYSTKDEFKSTAKKLGIMDDFKAGVPRVAYNGVV    420
C.gigas_X2_XP_034321805.1      LVKDTPEVTVSEAMSGHKSNMKALKIIYSTKDEFKSTAKKLGIMDDFKAGVPRVAYNGVV    411
C.gigas_X3_XP_011453276.2      LVKDTPEVTVSEAMSGHKSNMKALKIIYSTKDEFKSTAKKLGIMDDFKAGVPRVAYNGVV    408
C.gigas_X4_XP_011453278.2      LVKDTPEVTVSEAMSGHKSNMKALKIIYSTKDEFKSTAKKLGIMDDFKAGVPRVAYNGVV    399
                               ************************************************************

C.gigas_X1_XP_034321804.1      SFMYRGQRIYLAPPSNWKGYDPKWS       445
C.gigas_X2_XP_034321805.1      SFMYRGQRIYLAPPSNWKGYDPKWS       436
C.gigas_X3_XP_011453276.2      SFMYRGQRIYLAPPSNWKGYDPKWS       433
C.gigas_X4_XP_011453278.2      SFMYRGQRIYLAPPSNWKGYDPKWS       424
                               *************************
```

Fig.S2 Sequence comparison of the GnT-I isoforms X1-X4 from *C. gigas*

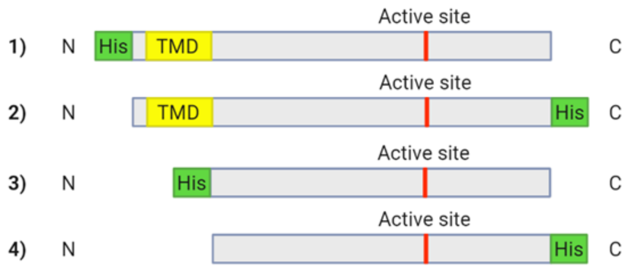

Fig.S3 Schematic structure of the full-length (1 and 2) and truncated (3 and 4) recombinant constructs of GnT-I from C. gigas (XP_034321804.1). Only the full-length constructs contain the transmembrane domain (yellow). All constructs contain a N- or C-terminal 6x His-tag (green) for purification. Red line marks the active site D289 (proton acceptor)

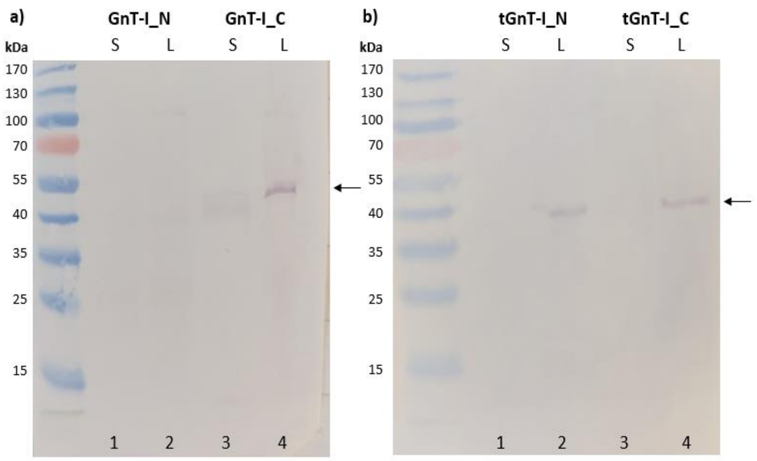

Fig.S4 Expression of the recombinant GnT-I from *C. gigas* with N- or C-terminal 6xHis-tag. (a) Full length GnT-I enzyme (~52 kDa). 1 = supernatant GnT-I_N, 2 = lysate GnT-I_N, 3 = supernatant GnT-I_C, 4 = lysate GnT-I_C. (b) Truncated GnT-I enzyme without transmembrane domain (~50 kDa). 1 = supernatant tGnT-I_N, 2 = lysate tGnT-I_N, 3 = supernatant tGnT-I_C, 4 = lysate tGnT-I_C. Supernatant represents the liquid portion of the cell culture medium after centrifugation and contains secreted proteins. Lysate contains non-secreted proteins obtained by cell disruption.
